# Energy Conversion Capacity of Barium Zirconate Titanate

**DOI:** 10.3390/ma13020315

**Published:** 2020-01-09

**Authors:** Nawal Binhayeeniyi, Pisan Sukwisute, Safitree Nawae, Nantakan Muensit

**Affiliations:** 1 Faculty of Science and Technology, Princess of Naradhiwas University, Narathiwat 96000, Thailand; safitree.n@pnu.ac.th; 2 Department of Physics, Faculty of Science, King Mongkut’s Institute of Technology Ladkrabang, Bangkok 10520, Thailand; pisan.su@kmitl.ac.th; 3 Department of Physics, Faculty of Science, Prince of Songkla University, Songkhla 90110, Thailand; nantakan.m@psu.ac.th; 4 Center of Excellence in Nanotechnology for Energy (CENE), Prince of Songkla University, Songkhla 90110, Thailand

**Keywords:** lead-free ceramic, sol–gel process, barium zirconate titanate, dielectric property

## Abstract

In this study, we investigated the effect of zirconium content on lead-free barium zirconate titanate (BZT) (Ba(Zr*_x_*Ti_1−*x*_)O_3_, with *x* = 0.00, 0.01, 0.03, 0.05, and 0.08), which was prepared by the sol–gel method. A single-phase perovskite BZT was obtained under calcination and sintering conditions at 1100 °C and 1300 °C. Ferroelectric measurements revealed that the Curie temperature of BaTiO_3_ was 399 K, and the transition temperature decreased with increasing zirconium content. At the Curie temperature, Ba(Zr_0.03_Ti_0.97_)O_3_ with a dielectric constant of 19,600 showed the best performance in converting supplied mechanical vibration into electrical power. The experiments focused on piezoelectric activity at a low vibrating frequency, and the output power that dissipated from the BZT system at 15 Hz was 2.47 nW (30 MΩ). The prepared lead-free sol–gel BZT is promising for energy-harvesting applications considering that the normal frequencies of ambient vibration sources are less than 100 Hz.

## 1. Introduction

Lead zirconate titanate (PZT, Pb(Zr*_x_*Ti_1−*x*_)O_3_), a lead-based material with a high piezoelectric coefficient and electromechanical coupling factor, is one of the most promising materials for use in fabricated energy-harvesting devices [1,2,3] because its perovskite structure exhibits dielectric, ferroelectric, and piezoelectric properties [2,3]. However, PZT is toxic to the environment. Therefore, innovative lead-free dielectric materials with piezoelectric properties have been formulated to address this environmental issue. Among these materials is BaTiO_3_, which is a well-known material possessing a perovskite structure with high dielectric properties, a low dielectric loss tangent, and dielectric reliability [4,5,6,7].

BaTiO_3_ can be modified by doping it with additives such as Sr^2+^, Ca^2+^, Sn^4+^, and Zr^4+^ [4]. Doping BaTiO_3_ with ZrO_2_ can improve the dielectric and piezoelectric properties because the chemical stability of Zr^4+^ is greater than that of Ti^4+^ [4,5,6,7]. In addition, the Curie temperature also changes; that is, it decreases as the Zr content increases [5,6,7,8].

BaTiO_3_ can be used in tunable capacitor devices and dynamic random-access memory applications. Moreover, it is also applied in actuators and energy storage devices because the strain that is induced by the electric field retains dipole moment behavior and energy storage properties [7,9,10]. Lui et al. prepared BaTi_0.7_Zr_0.3_O_3_ ceramic by spark plasma sintering. The maximum energy storage density of the ceramic was determined to be 0.51 J/cm^3^ [9]. Moreover, Puli et al. investigated the energy storage of barium calcium titanate (BCT) ceramic and obtained a high energy density (0.24 J/cm^3^) [10].

There are various kinds of energy harvesters, including thermoelectric, electromagnetic, electrostatic, and piezoelectric. Of these methods, piezoelectric energy harvesting is very attractive for the system’s small size, high output power, and ease of operation [11,12,13].

In this study, we investigated the crystal structure, dielectric properties, phase transition, and the degree of diffuseness of lead-free barium zirconate titanate (BZT) ceramics with various Zr contents. Additionally, the energy conversion behavior resulting from the modification of BZT was examined. These materials might lead to a reduction in the use of the lead-based bulky ceramics that are usually required in applications.

## 2. Materials and Methods

Ba(Zr*_x_*Ti_1−*x*_)O_3_ (*x* = 0.00, 0.01, 0.03, 0.05, and 0.08) was prepared by the sol–gel method. Barium acetate (HIMEDIA^®^, Mumbai, MH, India, 99.0%), zirconium(IV) propoxide (Sigma-Aldrich^®^, St. Louis, MO, USA, 70 wt.% in 1-propanol), and titanium(IV) isopropoxide (Sigma-Aldrich^®^, St. Louis, MO, USA, were used as the reagents. Glacial acetic acid (Merck, Darmstadt, HE, Germany, 100%) and 2-methoxyethanol (Ajex Finechem Pty Ltd, Taren Point, NSW, Australia) were used as solvents in the sol–gel method following Jiwei et al. [14]. The procedure has been reported elsewhere [15,16]. The gels were dried in an oven for 24 h. All dried gels were calcined at 1100 °C for 2 h in alumina crucibles. The BZT powder was ball-milled in ethanol milling media (Merck, Ethanol Absolute, Darmstadt, HE, Germany) for 24 h (200 rpm) by using a high-energy planetary ball mill (Retsch PM100, Haan, NW, Germany). The milled powders were blended with a small amount of polyvinyl alcohol (PVA) to form discs (diameter 13 mm) at 100 MPa. All the green bodies were sintered at 1300 °C for 2 h in closed alumina crucibles. The upper and lower surfaces of the sintered ceramics were covered by silver paste and then calcined at 600 °C for 0.5 h for use as electrodes for the dielectric measurements.

The dielectric properties and ferroelectric phase transitions of all samples were characterized at 25–150 °C (at 1 kHz) by an LCR meter (Hewlett Packard 4263 B, Mississauga, ON, Canada). The crystalline structure of BZT was determined by X-ray diffraction (XRD, PHILLIPS X’pert MPD, Almelo, OV, Netherlands) with Ni-filtered CuK_α_ radiation. The XRD analysis was performed at room temperature (20° ≤ 2θ ≤ 77°) with a step size of 0.02°. The bulk densities of the sintered BZT discs were measured in accordance with the Archimedes method. Thermal analysis of the dried BZT gels was performed by differential thermal analysis (DTA, Perkin Elmer DTA7, Norwalk, CT, USA) and thermogravimetric analysis (TGA, Perkin Elmer TGA7, Norwalk, CT, USA). The thermal analysis results were collected from 50 °C to 1300 °C at a rate of 10° C/min. Surface microstructures were observed using scanning electron microscopy (SEM, quanta400, Thermo Fisher Scientific, Brno, JM, Czech Republic) with an accelerating voltage of 20 kV and 3000× magnification. The grain sizes were analyzed by averaging over the total number of grains in the SEM images.

## 3. Results and Discussion

**Figure 1:**
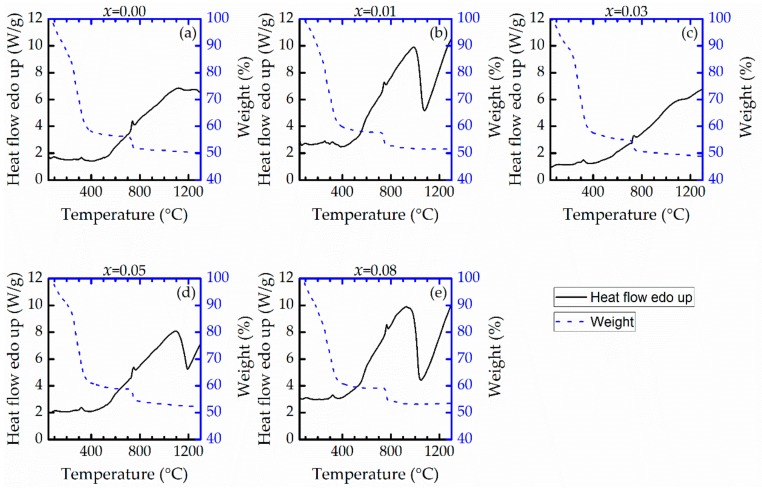
TGA and DTA plots for Ba(Zr*_x_*Ti_1−*x*_)O_3_ samples with *x* composition of (**a**) 0.00, (**b**) 0.01, (**c**) 0.03, (**d**) 0.05 and (**e**) 0.08 mol.

**Figure 2:**
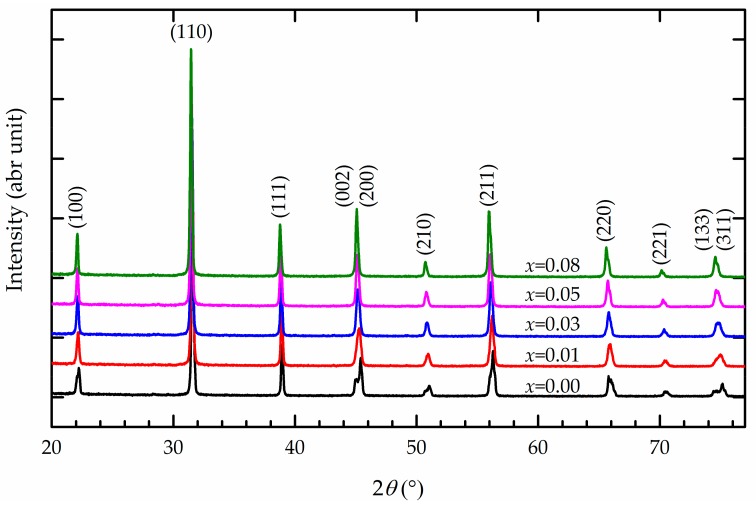
XRD patterns of the Ba(Zr*_x_*Ti_1−*x*_)O_3_ ceramics.

**Figure 3:**
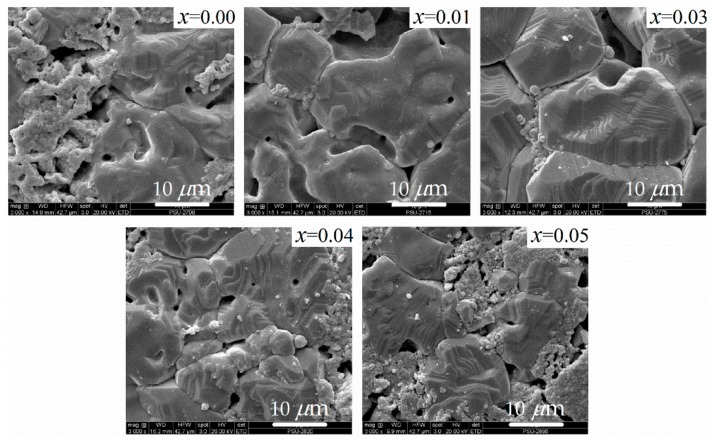
SEM images of the sintered Ba(Zr*_x_*Ti_1−*x*_)O_3_ ceramics.

The TGA and DTA results in Figure 1 show three mechanisms. First, the endothermic reaction observed in the temperature range of 25–200 °C is associated with the dehydration of the dried BZT gels, as observed by the mass loss of about 20%. Second, in the temperature range of 200–650 °C, a major mass loss occurs with the emission of CO_2_, solvents, and organic compounds because of the thermal disintegration of the polymeric dried gels and primary synthesis of Ba(Zr*_x_*Ti_1−*x*_)O_3_ via BaCO_3_–TiO_2_ and BaCO_3_–ZrO_2_ core–shell particles [17,18,19]. Third, the exothermic peak in the range of 650–1200 °C exhibits a slight weight loss that can be attributed to Ba(Zr*_x_*Ti_1−*x*_)O_3_ crystallization and the subsequent formation of the perovskite structure. This final mechanism is due to the decarbonation of BaCO_3_ to react with TiO_2_ and ZrO_2_. For these results, it is worth noting that although the calcination process is typically performed at temperatures as low as 650 °C, the calcination temperature used in this work was 1100 °C [15,16] to ensure the formation of the pure perovskite structure of Ba(Zr*_x_*Ti_1−*x*_)O_3_ without secondary phases, as seen in the following XRD result (Figure 2). Calcination at a temperature above 1100 °C should not be undertaken, because of the agglomeration and enlargement of Ba(Zr*_x_*Ti_1−*x*_)O_3_ particles. The compression of large calcined particles might result in a low bulk density of the sintered ceramics [20,21]. Table 1 presents the measurable bulk density of sintered Ba(Zr*_x_*Ti_1−*x*_)O_3_. The relative density of all samples is 93.5% ± 0.21%. The addition of zirconium does not affect density [8]. A sintering temperature of 1300 °C is sufficient to fuse the as-calcined Ba(Zr*_x_*Ti_1−*x*_)O_3_ powders, and a calcination temperature of 1100 °C has an insignificant effect on the bulk density. The XRD patterns of sintered Ba(Zr*_x_*Ti_1−*x*_)O_3_ ceramics (with *x* = 0.00, 0.01, 0.03 0.05, and 0.08) are shown in Figure 2. The structure of all Ba(Zr*_x_*Ti_1−*x*_)O_3_ ceramics is a pure perovskite phase without an impurity phase. With the addition of Zr, the peak shifts to a lower angle because the ionic radius of Zr^4+^ (0.079 nm) is larger than that of Ti^4+^ (0.068 nm) [5]. It is clear that the tetragonal phase of BaTiO_3_ ceramic is characterized by the splitting of the (0 0 2) and (2 0 0) diffraction peaks at 44.93° and 45.40°, respectively (the calculated values of the cell parameters of BaTiO_3_ are (a ~ 3.9906 Å, c ~ 4.0301 Å), respectively). As the zirconium content increases, the two diffraction peaks merge into one peak. This corresponds with the change in the structure of the BZT system from tetragonal to orthorhombic at room temperature, as previously reported by [4,6,22]. According to, the separation of (1 3 3) and (3 1 1) diffraction peaks of Ba(Zr_0.03_Ti_0.07_)O_3_ occurs at diffraction angles of 74.63° and 74.91°, respectively; upon the addition of 5 mol.% zirconium content, a single diffraction peak is observed. This is caused by the structure transforming from orthorhombic to rhombohedral [4,6]. It is concluded that the increased zirconium content changes the structure of BZT ceramic from tetragonal to rhombohedral, which is confirmed by the gradual merging of XRD peaks [6,8,21]. Finally, the dense ceramic discs exhibit large grains and a small proportion of fine grains with pores. The grains are irregular in shape, with an average grain size of 10–30 µm, because the initial size of the powder is changed by the ball milling process [20,21], as shown in Figure 3.

The relative permittivity (*ε*_r_) or dielectric constant and dielectric loss (tan δ) at *T_m_* (1 kHz) are listed in Table 1. The dielectric constant increases with zirconium content until it reaches 3 mol.%. Ba(Zr_0.03_Ti_0.07_)O_3_ ceramic has the highest dielectric constant, which is reduced when zirconium reaches 5 mol.%. The dielectric loss of all BZT ceramics depends on the zirconium content and ranges from 0.072 to 0.0392, similar to the results of our previous work [15].

**Figure 4:**
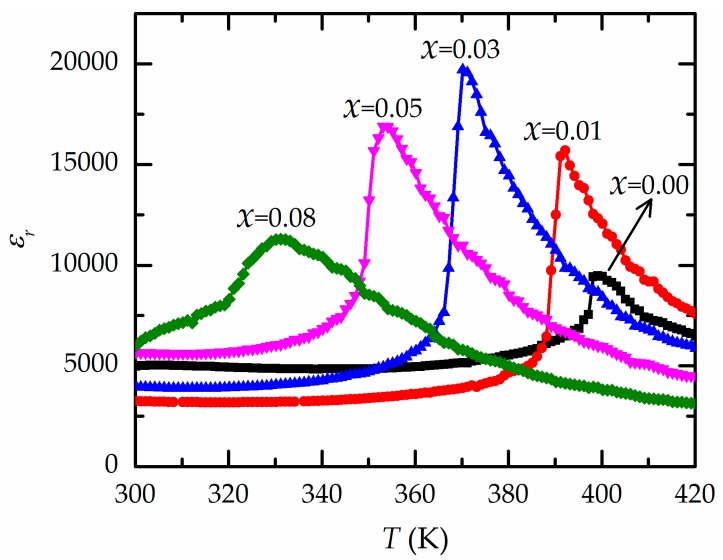
Relationships between the dielectric constant (*ε*_r_) and temperature for all samples of Ba(Zr*_x_*Ti_1−*x*_)O_3_ ceramics.

Figure 4 presents the values of the relative permittivity (*ε*_r_) or dielectric constant measured at 1 kHz for the Ba(Zr*_x_*Ti_1−*x*_)O_3_ samples. The position of each dielectric peak moves to a higher temperature with the addition of Zr, which ranges from 0 to 3 mol.%. For *x* = 0.08, the dielectric peak is broad because of the inhomogeneous distribution of Zr^4+^ ions in the Ti sites and the non-uniform stress in the grains [8,23]. The highest dielectric constant is 19,600 for Ba(Zr_0.03_Ti_0.97_)O_3_. Further increases in Zr content cause a decrease in the temperature *T_m_* with the maximum dielectric value (Table 1), as described in the literature [6,8]. This is the result of the increased substitution of the Zr^4+^ ion in the B sites of BaTiO_3_, causing a change in the *d*-spacing of the Ba(Zr*_x_*Ti_1−*x*_)O_3_ structure [6,16] and resulting in a decrease in the phase transition temperature or *T*_m_ [8,22]. For low Zr content (*x* < 0.15), at the apex of the dielectric curve, *T*_m_ can be considered the Curie temperature (*T*_c_) [21]. A rapid increase in the *ε*_r_ value occurs near *T*_c_ because the BZT structure is thermally excited to a tetragonal–cubic intermediate phase (ferroelectric–paraelectric phase transition) when the temperature changes to *T*_m_. This results in a large degree of unstable polarization, and consequently, an applied electric field can easily produce considerable variation in polarization [24]. The decrease in the dielectric constant above *T*_c_ is caused by the thermal detriment of polarization alignment [24,25].

**Figure 5:**
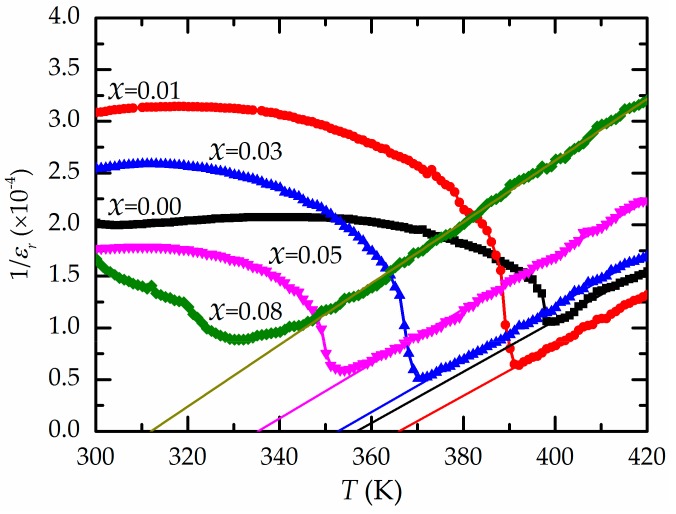
Relationships between the temperature and the inverse dielectric constant at 1 kHz for all the Ba(Zr*_x_*Ti_1−*x*_)O_3_ ceramics.

Because BZT ceramic is classified as a ferroelectric material, the dielectric characteristic of BZT above the Curie temperature corresponds to the Curie–Weiss law: 1/*ε*_r_ = (*T* *− T*_0_)/*C* (*T* > *T*_c_), where *T*_0_ and *C* are the Curie–Weiss temperature and constant, respectively. For all analyzed BZT compositions, the inverse *ε*_r_ versus temperature data were fitted to the Curie–Weiss law, as shown in Figure 5. The *T*_0_ fitting parameters are listed in Table 1. It is clear that the reciprocal *ε*_r_ value follows the Curie–Weiss law for *T* > *T*_m_. The divergence of the reciprocal *ε*_r_ value from the Curie–Weiss law is defined as ∆*T*_m_ = *T*_cw_ − *T*_m_, where *T*_cw_ is the temperature at which the value of the reciprocal *ε*_r_ value begins to diverge from the Curie–Weiss law. From the results in Table 1, the ∆*T*_m_ value increases with Zr content because of the influence of the Zr^4+^ ions on the shift in the ferroelectric–paraelectric transition temperature of BZT [5,6,26].

**Figure 6:**
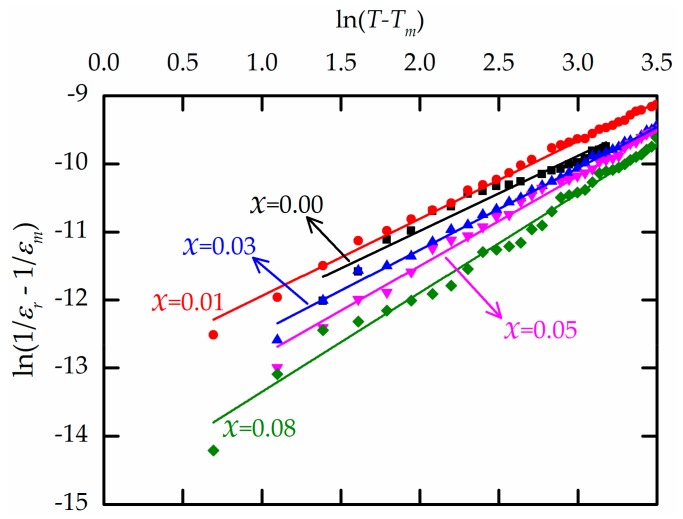
Linear relationships between ln(1/*ε*_r_ − 1/*ε*_m_) and ln(*T* − *T*_m_) for all *x* values.

The degree of diffuseness of the phase transition can be formulated by a modified Curie–Weiss law [27]:

(1) $$\frac{1}{\varepsilon_{r}}-\frac{1}{\varepsilon_{m}}=\frac{{\left(T-T_{m}\right)}^{\gamma }}{C^{*}}\left(1\leq \gamma \leq 2\right),$$

where *γ* and C^*^ are constants derived from fitting the experimental data. The *γ* value provides information about the behavior of ferroelectric materials. For a normal ferroelectric, *γ* = 1. For an ideal ferroelectric relaxor associated with quadratic dependence, *γ* = 2. Figure 6 shows the plot of ln(1/*ε_r_* − 1/*ε_m_*) against ln(*T* − *T_m_*) at 1 kHz. The fitted *γ* values (Table 1) show that the higher the Zr content, the higher the diffuse phase transition, as reported in previous works [6,8]. Consequently, the inclusion of the diffusive Zr^4+^ ion in the octahedral site of the perovskite structure causes the common ferroelectric to transform into a ferroelectric relaxor [9,28]. The dielectric losses of all the BZT ceramics range from 1% to 5%. It is clear that the dielectric losses show the same trends with increasing temperature.

Each sample was investigated for the capability of energy conversion, as described by Sukwisute et al. [1]. Each disc (thickness ~ 1 mm) was rigidly glued onto a vibrating structure at a constant operating frequency of 15 Hz. Varying resistors were connected to each disc, and the potential in the circuit was measured to calculate the output power according to P_ac_ = V^2^/R, where *V* is the potential and *R* is the resistance. The calculated values are summarized in Table 2. The Ba(Zr_0.03_Ti_0.97_)O_3_ ceramic shows the capability of energy conversion of the supplied mechanical vibration to electrical power. This is attributed to the highest dielectric constants and the transformation of the common ferroelectric to a relaxor ferroelectric, as reported previously [9,28]. In previous work, Rukbamrung et al. used the standard harvesting approach to determine the energy-harvesting ability of PZT + 1 mol.% Mn and PMN-25PT, and they obtained a power of 1.7 and 4.5 µW [2]. The BZT ceramics in our study can be operated at the low frequencies used in daily activities, such as walking and running. In addition, the normal frequencies of ambient vibration sources are much less than 100 Hz [11,12]. From this practical viewpoint, BZT ceramic can be very useful in low-frequency energy-harvesting devices.

## 4. Conclusions

Ba(Zr*_x_*Ti_1−*x*_)O_3_ ceramics with various zirconium contents (*x* = 0.00, 0.01, 0.03, 0.05, and 0.08) were produced by the sol–gel method. A single-phase perovskite BZT was obtained under calcination and sintering conditions at 1100 °C and 1300 °C. All BZT samples had a pure perovskite structure without a secondary phase. The crystal structure changed with the zirconium content. Ferroelectric measurements of the ceramics showed that the Curie temperature of BaTiO_3_ was 399 K, and further increases in the zirconium content decreased the Curie temperature to 331 K. At the phase transition, Ba(Zr_0.03_Ti_0.97_)O_3_ had the highest dielectric constant of 19,600 and exhibited good performance in converting supplied mechanical vibration to electrical power. Thus, Ba(Zr_0.03_Ti_0.97_)O_3_ is promising for mechanical energy-to-electrical energy coupling at low frequencies, with no damage observed at high temperatures.

## Figures and Tables

**Table 1:** Relative density, the values of the dielectric constant (*ε*_r_) at *T_m_* (1 kHz), dielectric loss (tanδ) at *T_m_* (1 kHz), Curie–Weiss temperature (*T*_0_), Curie–Weiss constant (*C*), Curie–Weiss law temperature (*T_cw_*), *T_m_*, Δ*T_m_*, and *γ* for all *x* values of Ba(Zr*_x_*Ti_1−*x*_)O_3_.

Ba(Zr*_x_*Ti_1−*x*_)O_3_	Relative Density (%)	*ε*_r_ at *T_m_* (1 kHz)	tanδ at *T_m_* (1 kHz)	*T*_0_ (K)	*C* (×10^5^ K)	*T_cw_* (K)	*T_m_* (K)	Δ*T_m_* (K)	*γ*
*x* = 0.00	93.26	9,496	0.0072	357	4.04	400	399	1	1.01
*x* = 0.01	93.66	15,702	0.0207	366	4.08	395	392	3	1.05
*x* = 0.03	93.76	19,698	0.0314	353	3.92	378	370	8	1.21
*x* = 0.05	93.49	16,891	0.0382	335	3.79	368	353	14	1.26
*x* = 0.08	93.32	11,294	0.0392	312	3.36	355	331	24	1.38

**Table 2:** Output power dissipated from the barium zirconate titanate (BZT) system.

Ba(Zr_x_Ti_1−x_)O_3_	V (±0.05 V)	R (MΩ)	P_ac_ (nW)
*x* = 0.00	0.24	132	0.044
*x* = 0.01	0.26	90	0.075
*x* = 0.03	0.86	30	2.47
*x* = 0.05	0.68	50	0.92
*x* = 0.08	0.28	90	0.09
